# Phenylpropanoid Defences in *Nicotiana tabacum* Cells: Overlapping Metabolomes Indicate Common Aspects to Priming Responses Induced by Lipopolysaccharides, Chitosan and Flagellin-22

**DOI:** 10.1371/journal.pone.0151350

**Published:** 2016-03-15

**Authors:** Msizi I. Mhlongo, Lizelle A. Piater, Ntakadzeni E. Madala, Paul A. Steenkamp, Ian A. Dubery

**Affiliations:** 1 Department of Biochemistry, University of Johannesburg, Auckland Park, Johannesburg, South Africa; 2 CSIR Biosciences, Natural Products and Agroprocessing Group, Pretoria, South Africa; University of Nebraska-Lincoln, UNITED STATES

## Abstract

Plants have evolved both constitutive and inducible defence strategies to cope with different biotic stimuli and stresses. Exposure of a plant to a challenging stress can lead to a primed state that allows it to launch a more rapid and stronger defence. Here we applied a metabolomic approach to study and compare the responses induced in *Nicotiana tabacum* cells by microbe-associated molecular pattern (MAMP) molecules, namely lipopolysaccharides (LPS), chitosan (CHT) and flagellin-22 (FLG22). Early response metabolites, extracted with methanol, were analysed by UHPLC-MS/MS. Using multivariate statistical tools the metabolic profiles induced by these elicitors were analysed. In the metabolic fingerprint of these agents a total of 19 cinnamic acid derivatives conjugated to quinic acids (chlorogenic acids), shikimic acid, tyramine, polyamines or glucose were found as discriminant biomarkers. In addition, treatment with the phytohormones salicylic acid (SA), methyljasmonic acid (MJ) and abscisic acid (ABA) resulted in differentially-induced phenylpropanoid pathway metabolites. The results indicate that the phenylpropanoid pathway is activated by these elicitors while hydroxycinnamic acid derivatives are commonly associated with the metabolic response to the MAMPs, and that the activated responses are modulated by both SA and MJ, with ABA not playing a role.

## Introduction

Plants are continuously exposed to a vast array of biotic stresses that affect their vigour and survival [1,2]. Their close association with different organisms in the environment ranges from pathogenic to beneficial [3]. To defend themselves, plants use preformed defence mechanisms which prevents both pathogen entrance and herbivore feeding. Following attempted pathogen infection, however, plants activate microbe-associated molecular pattern (MAMP)-triggered immunity (MTI) which relies on the detection of non-self conserved molecules [1,2,4–6]. The ability of a potential pathogen to cause disease is closely related to the host’s ability to recognise and respond to these non-self entities from the pathogen [7]. Pre-exposure of a plant to MAMPs can induce a physiological change to allow the plant to respond faster and stronger to subsequent pathogen attacks [8,9].

Phytohormones such as abscisic acid (ABA), salicylic acid (SA), ethylene (ET) and jasmonates (JA) play important roles in plant responses to environmental stresses [2,10–12]. Upon infection, phytohormones accumulate in varying amounts and this leads to reprogramming of the cell’s transcriptome, activation of defence genes and production of phytoalexins [2,13–15]. To minimise fitness cost of activating unnecessary defence genes and to specify the defence response, a mixture of hormones is produced that are specific to the stress detected. For example, SA-induced resistance is more effective against biotrophic pathogens [16] while JA- and/or ET-induced resistance is operative against nectrotrophic and herbivore attack [17]. Furthermore, different plant species can employ different signal transduction pathways to specify their immune response. These hormones interact either antagonistically or synergistically and modulate the reprogrammed defence output [2,18–20].

Recently, there has been renewed interest in utilising the activation of the innate immune system in plants as a tool in developing novel crop protection strategies [21]. In this context it has been reported that plant treatment with immune-inducing / -boosting agents results not only in strong and fast immune responses but also in the induction of resistance to a number of environmental stresses. This form of induced and enhanced immunity has been described as plant priming, pre-conditioning or sensitisation [8,22,23]. Extensive transcriptomic and proteomic studies to investigate the action mechanisms of priming agents derived from pathogens have been performed and different phytohormone-dependent signalling pathways have been reported for some priming agents [18,24,25], suggesting that MAMPs act *via* that hormone-dependent pathway. In contrast, there are few related reports on priming mechanisms at the metabolome level [9]. Metabolites, as the final products of biological information flow [26], act as regulatory components and thus allow for a more complete picture of the physiological state of a plant [27]. Using a similar approach, we aimed to investigate if such conclusions can be made at a metabolic level. Here, using metabolite profiling, metabolites found to be induced by pathogen-derived agents were compared to those induced by defence-associated phytohormones. The results are discussed against the background of recent developments in the knowledge regarding priming.

## Materials and Methods

### Elicitation of cell cultures and metabolite extraction

Three days after subculturing, tobacco (*Nicotiana tabacum* cv. Samsun) cell suspensions grown in Murashige and Skoog (MS) medium [28,29], were treated with pathogen-derived elicitors at 100 μg/mL concentrations for LPS and CHT and 200 nM for FLG22, while phytohormone concentrations were 0.1 mM for ABA, 0.2 mM for MJ and 0.3 mM for SA. Following elicitation, cells were harvested at different time intervals (0, 6, 12, 18 and 24 h), collected by filtration and washed with sterile MS medium.

Two (2) g cells were transferred to 50 mL Falcon tubes and homogenised with 20 mL 100% methanol (ratio 1:10 m/v) using a probe sonicator (Sonopuls, Bandelin, Germany) set at 55% power for 15 sec, repeated twice. Cell debris was pelleted by centrifugation at 5000 rpm in a benchtop centrifuge and the supernatants transferred to new sterile 50 mL Falcon tubes. The supernatants were concentrated to approximately 1 mL using a Büchi rotary evaporator at 55°C. These were transferred to 2 mL Eppendorf centrifuge tubes and evaporated in a heating block at 55°C overnight to complete dryness. The resulting dry pellets were then redissolved in 400 μL 50% methanol (mass spectrometry-grade methanol and milliQ water), filtered through a 0.22 μm nylon filter into glass vials fitted with 500 μL inserts and stored at -20°C until analysis.

For data reproducibility, the experimental design comprised of three biological repeats and three analytical replicates (n = 9).

### Ultra high performance liquid chromatography—mass spectrometry

Data acquisition of these methanol extracts was performed on a UHPLC-high definition quadrupole time-of-flight MS instrument (UHPLC-QTOF SYNAPT G1 HD-MS system, Waters Corporation, Manchester, UK) fitted with a T3 Acquity column (1.7 μm, 2.2 mm X 150 mm; Waters Corporation, Manchester, UK) using a binary solvent gradient of 0.1% formic acid in water (solvent A) and 0.1% formic acid in acetonitrile (Romil, Cambridge, UK) (solvent B). The gradient was set as follows: 5% B over 0.0–2.0 min, 5–12% B over 2.0–2.10 min, 12–65% B over 2.10–10.50 min, 65–95% B over 10.50–11.00 min, held constant at 95% B over 11.00–12.00 min, and returning from 95–5% B over 12.00–13.00 min. The column was washed with 5% B over 13.00–15.00 min to return to the initial conditions. The PDA detector scanning range was from 200–500 nm with 1.2 nm resolution and a sampling rate of 20 points/sec.

The MS detector was set to collect both negative and positive ionisation data, however, only the negative data is presented here. The condition of the MS detector was as follows: capillary voltage: 2.5 kV, sample cone voltage: 30 V, microchannel plate (MCP) detector voltage: 1600 V, source temperature: 120°C, desolvation temperature: 400°C, cone gas flow: 50 L/h, desolvation gas flow: 800 L/h, *m/z* range: 100–1000, scan time: 0.15 s, interscan delay: 0.02 s, mode: centroid, lockmass: leucine enkephalin (556.3 g/mL), lockmass flow rate: 0.4 mL/min, mass window: 0.5 Da. Samples were analysed in a randomised manner and pooled samples were included to monitor analytical reproducibility and sample stability.

To assist with the downstream annotation and identification of the biomarkers associated with these treatments, the MS experiment file was setup to perform unfragmented as well as five fragmenting experiments (MS^E^) simultaneously. Ion fragmentation was performed at increasing the in-source collision energy (3 eV–30 eV).

### Multi-variate data analysis (MVDA)

The acquired data was analysed by SIMCA-13.0 (Soft independent modelling of class analogy) software (Umetrics Corporation, Umea, Sweden) and XCMS online (https://xcmsonline.scripps.edu) in order to maximise the identification of biomarkers associated with the different treatments. First the data was processed with MassLynx XS^™^ software (Waters, Manchester, UK) for alignment, peak finding, peak integration and retention time (Rt) correction with the following parameters: Rt range of 2.5–11 min, mass range of 100–1000 Da, mass tolerance of 0.02 D, Rt window of 0.2 min. Data was normalised to total intensity (area) using MarkerLynx XS^™^. The dataset obtained from MarkerLynx processing was exported to the SIMCA 13.0 software in order to perform principal component analysis (PCA) and orthogonal projection to latent structures discriminant analysis (OPLS-DA) modelling, and *Pareto* scaling was used for both models. PCA and OPLS-DA score plots were used to visualise and explain the metabolic differences between the samples. The generated models were evaluated by ‘metabolomic diagnostics tools’ namely the cumulative model variation in the matrix X, goodness-of-fit parameter (R^2^X(*cum*)), the proportion of the variance of the response variable that is explained by the model, R^2^Y(*cum*) and predictive ability parameter (R^2^Y(*cum*)), also known as the total variation fraction of matrix X predicted by an extracted components [30,31]. The OPLS-DA was further validated using CV-ANOVA (analysis of variance testing of cross-validation predictive residuals), where a *p*-value of < 0.05 is an indication of a good model [32]. Metabolites which were affected by the treatments were highlighted as discriminatory biomarkers by the PCA loading plots and OPLS-DA S-plots, where in the OPLS-DA S-plot only significant metabolites with the correlation [*P(corr)*] of ≥ 0.6 and covariance of (p1) ≥ 0.5 were chosen for metabolite identification using their *m/z* to generate elemental composition.

Secondly, MassLynx raw data (.raw) was converted using the DataBridge software (Waters, MA, USA) to NetCDF files. These were exported to the XCMS online statistical package (https://xcmsonline.scripps.edu), an automated, web-based metabolomics data processing software that identifies biomarker features of which the relative intensity varies between sample groups, for MVDA. The software further calculates the *p*-values as well as fold-changes of the metabolites (variables) across different samples of varying biological background. The method parameters were chosen for UHPLC/UHD-QTOF specificities and were as follows: (i) feature detection set as centWave method, minimum peak width = 5, maximum peak width = 20, (ii) Rt correction set as Obiwarp method, Profstep = 1, alignment set as *m/z* width = 0.015, minfraction = 0.5, bw = 5, and statistics set as statistical test = Unpaired parametric t-test (Welch t-test), paired t-test and post-hoc analysis with the threshold *p*-value = 0.01 and fold-change = 1.5. Upon completion of the XCMS analyses, PCA score and Cloud plots were generated to detect major differences between control and treated samples.

### Annotation of biomarkers

Annotation of chlorogenic acids and related cinnamic acid derivatives extracted from the elicited cell cultures was with the aid of UHPLC-QTOF-MS/MS based on the in-source collision-induced dissociation (ISCID) method as previously reported [33]. Accurate mass MS-based compound annotation was used. Single ion extracted chromatograms were extracted for each significantly induced ion, and its spectral fragments were compared among the different collision energies and with available spectral information. The mass spectrum of the extracted ion peak was used to deduce the putative empirical formula of the compound. Databases such as Dictionary of Natural Products (www.dnp.chemnetbase.com) and ChemSpider (www.chemspider.com) were consulted for the compound identity search. Additionally, the mass spectra were compared to published information on MS-based compounds annotation. Due to the lack of authentic standards, annotations represent putative identifications with assigned features at a metabolite identification (MI) level-2 annotation [34].

## Results and Discussion

**Fig 1:**
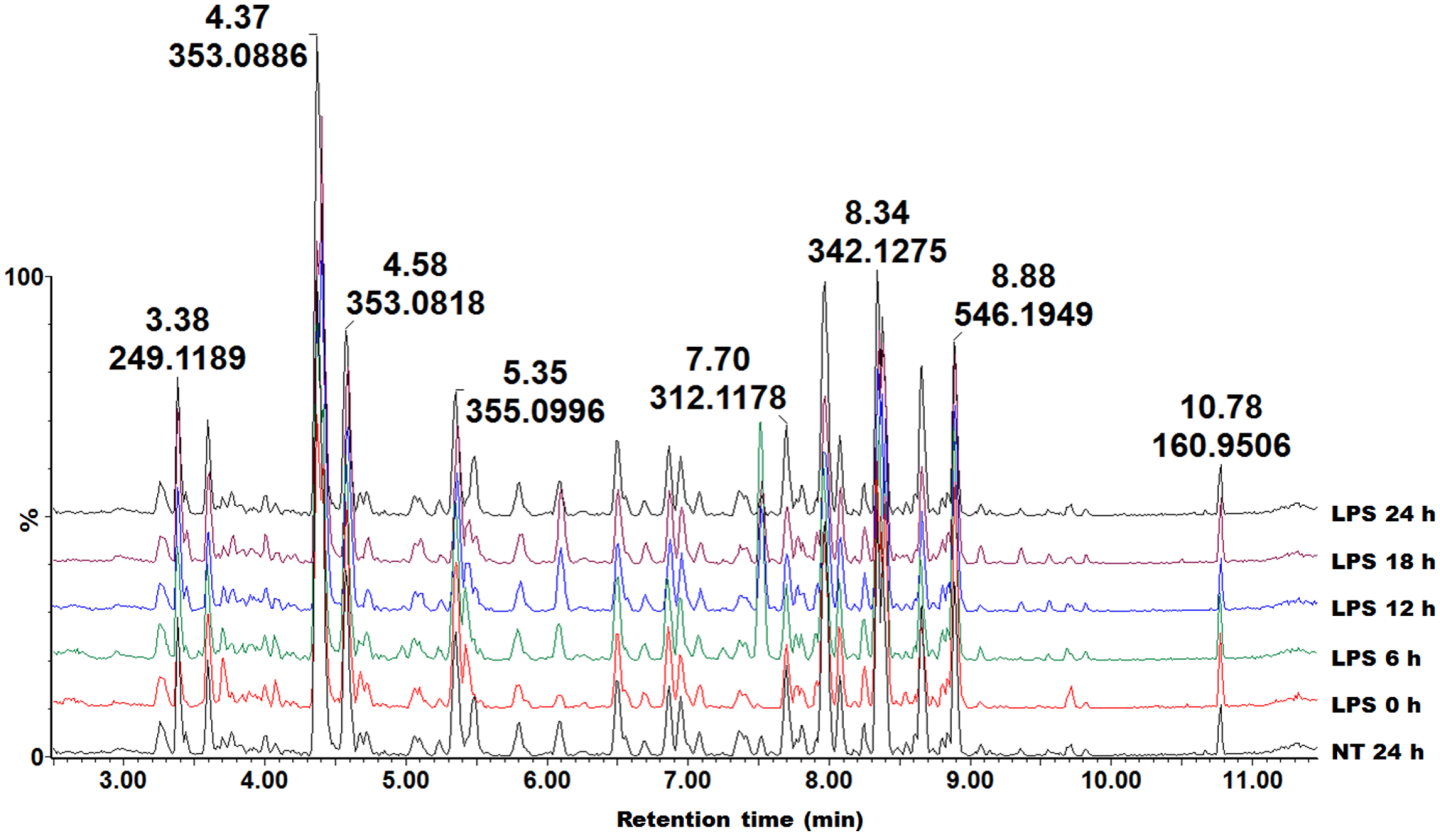
UHPLC-MS BPI chromatograms (ESI^-^) of methanol extracts of tobacco cells treated with LPS. The extracts were prepared from cells harvested at different time intervals (0–24 h). NT 24 h is a non-treated sample incubated for 24 h. The chromatograms show time-dependent changes, reflecting metabolic changes over time.

There is a strong body of evidence supporting a role for secondary metabolites as crucial determinants of plant resistance against disease. Hence, it was attempted to find a link between the molecules induced by MAMPs and those by hormones as signalling molecules, and to investigate to what extent the selected MAMPs act through convergent signalling mechanisms [35]. UHPLC-QTOF-HDMS was chosen as a preferred analytical platform for the analysis of the mostly polar metabolites extracted with methanol [33]. Base peak intensity (BPI) chromatograms (e.g. Fig 1) of the cell suspension extracts treated with different agents showed qualitative and quantitative variations, accumulation of new peaks and disappearance of some. This is an indication that these agents altered the cellular metabolism, resulting in time-dependent and dynamic metabolic changes.

### Multivariate data analysis with SIMCA and XC-MS online

The acquired UHPLC-MS data was further analysed with multivariate statistical tools to find the signatory biomarkers associated with the different treatments. For all samples a pooled sample was included as a control to investigate the stability of the samples and was found to cluster around the 0: 0 coordinate on the PCA plots. MarkerLynx preprocessed data matrices (Rt, *m/z* and peak intensities) were exported to SIMCA 13.0 software for PCA and OPLS-DA analysis. PCA is a non-supervised model that provides a global visualisation of similarities and dissimilarities between (explained by PC1) and within (explained by PC2) the samples. Since PCA lacks predictive power, an alternative method, namely OPLS-DA, was chosen. By virtue of being a supervised method using only 2 conditions (pre-assigned as different), it is capable of extracting molecular differences in the samples investigated [26,36], and thus assists in the identification of features responsible for the observed differences. PCA and OPLS-DA are widely used tools, however, each has its own limitations. Hence in this study, XCMS online was used to complement the shortcoming of SIMCA 13.0 software and to aid in identifying more metabolites induced by the eliciting agents. Like OPLS-DA, XCMS uses two predefined conditions (treated and non-treated) to predict and analyse metabolic changes in the samples under study.

#### Principal component analysis

**Fig 2:**
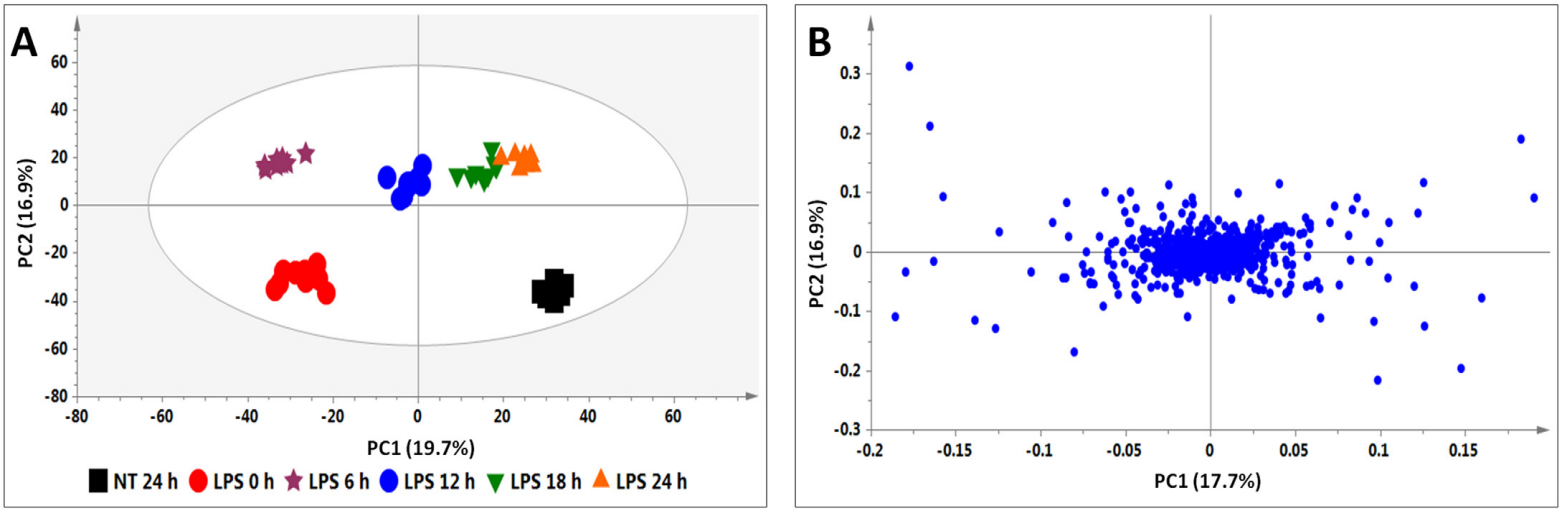
PCA analyses of the data from the LPS-treated cells. **(A)** PCA scores plot illustrating the different clustering of samples corresponding to the different time intervals. **(B)** The corresponding loadings plot showing discriminating variables responsible for the clustering observed in **(A)**. The ellipse represents Hoteling’s T2 at 95% confidence interval and the model calculated 4 PCs and gave R^2^X = 52.9% and Q_(cum)_ = 27.5%.

The PCA scores plot of the treated cells showed time-dependent clustering of the samples obtained from different time intervals, explaining the variation seen on the BPI chromatograms (e.g. Fig 2A). The corresponding loadings plots (Fig 2B) indicate the *m/z* ions responsible for the clustering on the PCA scores plots. The ions scattered furthest from the center are the ones responsible for the clustering observed on the PCA.

#### Orthogonal projection to latent structures discriminant analysis

**Fig 3:**
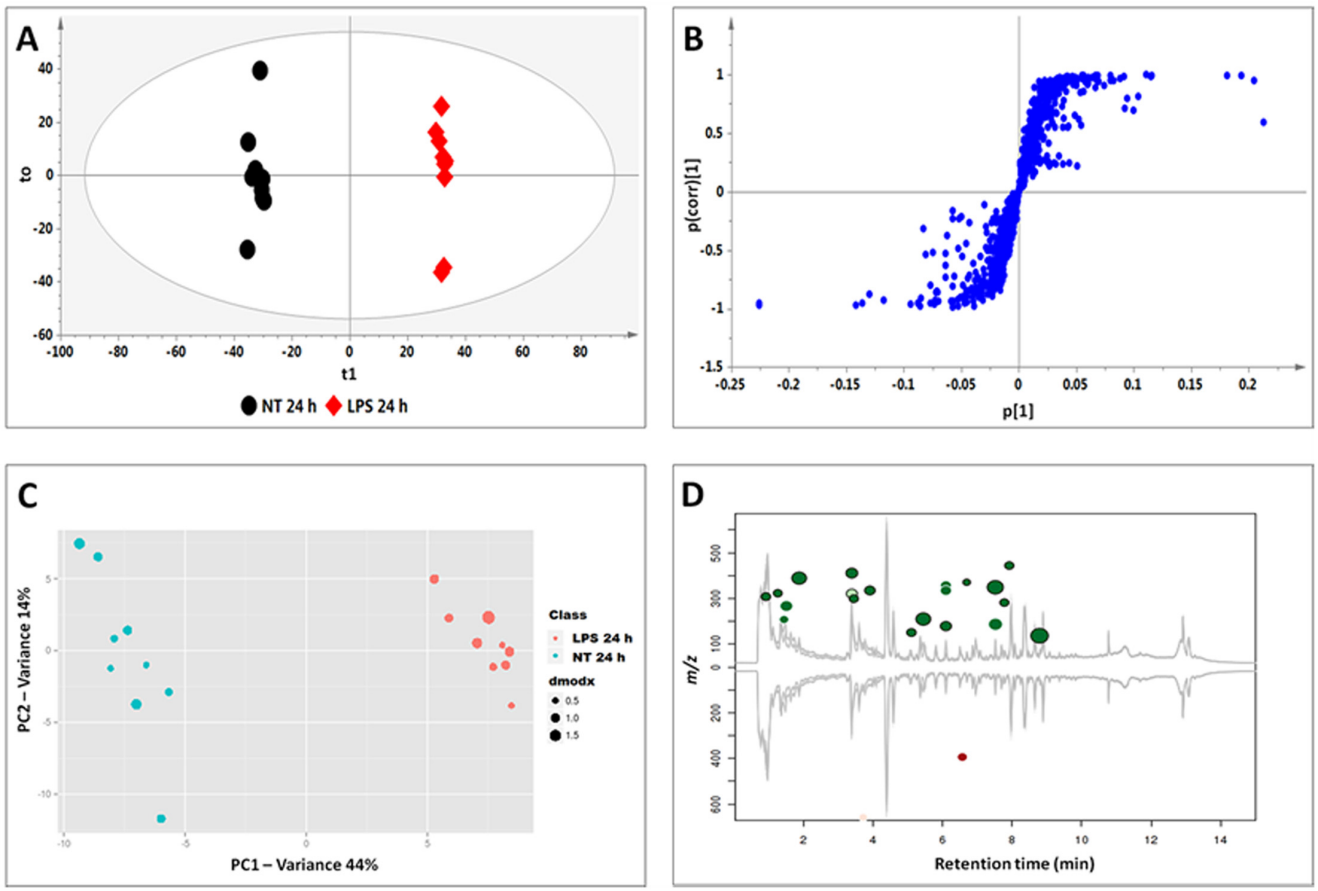
OPLS-DA (A and B) and XCMS (C and D) comparing the 24 h LPS-treated vs. 24 h non-treated cells. **(A)** The OPLS-DA scores plot and **(B)** the corresponding S-plot show the different clustering of treated and non-treated samples. The ellipse represents Hoteling’s T2 at 95% confidence interval and the model calculated PCs and gave R^2^X = 45.5%, R^2^Y = 99.7% and Q_(cum)_ = 98.5%. Model validation by CV-ANOVA showed high model significance with *p* value = 1.22 ×10^−11^. **(C)** The XCMA PCA scores plot of treated vs. non-treated cells processed by using unit variance scaling centered based on features intensity. **(D)** The Cloud plot of treated vs. non-treated cells showing data set of 35 features with *p*-value ≤ 0.01 and fold change ≥ 1.

The calculated OPLS-DA models (24 h non-treated and 24 h treated) also showed clear separation of the treated samples from the non-treated as seen on the OPLS-DA scores plots (e.g. Fig 3A). The corresponding S-plots (Fig 3B) allowed the prediction of significant features (potential biomarkers) of which the accumulation is related to the treatment.

#### XC-MS analysis

The 24 h non-treated and 24 h treated data set for all inducers was further investigated by XC-MS analysis. The PCA scores plot generated by the XC-MS (e.g. Fig 3C) show clear separation of the LPS-treated sample as seen on the OPLS-DA scores plot, thus also indicating metabolic differences in the compared samples. XCMS was chosen to complement SIMCA 13.0 since it offers other features that the latter does not have. The Cloud plot (Fig 3D) shows features of which the intensity increased on the upper plot in green, whereas features of which the intensity decreased are shown on the lower plot in red. The size of each bubble corresponds to the log fold-change of the feature: the larger the bubble, the larger the fold-change [37,38]. The statistical significance of the fold change, as calculated by a Welch t-test with unequal variances, is represented by the intensity of the feature’s colour where features with low *p*-values are brighter compared to features with high *p*-values [38–40]. The y-coordinate of each feature corresponds to the *m/z* ratio and the x-coordinate is the Rt of the compound. Each feature is also colour-coded such that features that are shown with a black border have database hits in METLIN (a metabolite mass spectral database, https://metlin.scripps.edu/), whereas features shown without a black border do not have database hits.

### Metabolome changes induced by MAMP agents (LPS, CHT and FLG22) in tobacco cells

When challenged by pathogens plants can trigger an immune response to arrest the onset of infection. This immune response is known to be based of the recognition of microbe-derived elicitors, *i*.*e*. MAMPs. The three MAMPs investigated; LPS, CHT and FLG22 are known to trigger defence responses in plants [41,42].

LPSs are Gram-negative bacterial cell membrane components found on the outer envelope where these lipoglycans contribute to survival by acting as a barrier between the environment and the bacterial cells. LPS plays a number of roles in the interactions between pathogens and eukaryotic cells. In plants LPSs are known to induce immune responses [43] and also implicated as systemic resistance inducers [44]. Chitosan (CHT) is a deacylated form of chitin containing poly-D-glucosamine found in fungal cell walls [42,45]. Earlier studies have shown that CHT is the most active compound in fungal cell walls, and is capable of inducing immune responses that directly inhibits fungal growth. These studies led to many others on chitosan as a plant protectant [42,46–48]. Flagellin is a major component of the flagellar filament in the bacterial flagellum and the FLG22 oligopeptide is a well characterised MAMP motif recognised by plants like *Arabidopsis thaliana* and *N*. *tabacum* [49,50].

**Fig 4:**
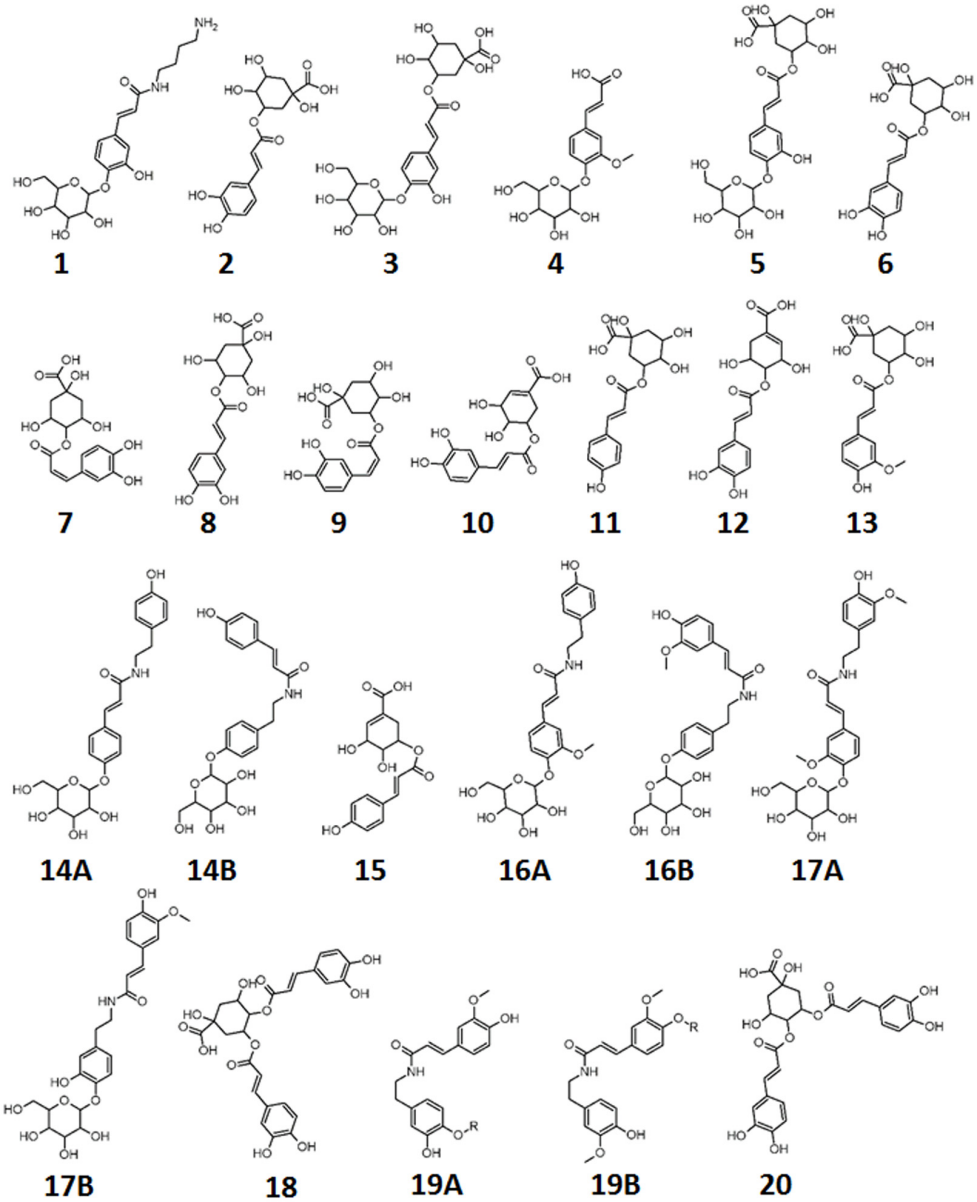
Structures of identified metabolites comprising the signatory biomarkers of the priming response of tobacco cells towards LPS, CHT and FLG. R represents unknown molecules attached to the identified: R = 204 Da.

Plant defence is a multi-layered mechanism with secondary metabolite production as one of the aspects. Using tobacco cells and the three MAMPs described above, we investigated the metabolic defences activated by these molecules. A total number of 19 cinnamic acid derivatives conjugated to quinic acids (chlorogenic acids), shikimic acid, tyramine, polyamines or glucose/hexose (Table 1 and Fig 4) was identified. Table 1 also serves to compare the metabolites perturbed by the different MAMP treatments.

**Table 1:** UHPLC-MS diagnostic ions (ESI-negative mode) used for the identification of biomarkers in LPS, CHT and FLG-treated tobacco cells. The common and unique biomarkers in response to MAMP elicitation are shown and compared to the phytohormones salicylic acid (SA), methyljasmonate (MJ) and absiscic acid (ABA). Biomarkers derived from both SIMCA and XC-MS analyses are listed. Annotated metabolites that were negatively correlated with the various treatments are indicated in italics and open circles.

Rt	*m/z*	Compound name	Diagnostic *m/z* fragment ions	CHT	FLG	LPS	SA	MJ	ABA
3.37	411.172	Caffeoylputrescine glycoside **(1)**	321.13, 249.12, 135.04	●	●		●	●	
3.59	353.086	3-Caffeoylquinic acid **(2)**	191.05, 179.05, 135.04	●	●	●	●		
3.81	515.141	3-O-(4’-O-Caffeoylglucosyl) quinic acid **(3)**	323.07, 191.06, 135.02	●					
4.06	355.092	Feruloylglycoside **(4)**	193.04					●	
4.17	515.142	5-O-(3’-O-Caffeoylglucosyl) quinic acid **(5)**	341.05, 179.04, 135.03	●					
4.33	353.085	*trans*-5-Caffeoylquinic acid **(6)**	191.05, 135.04	●	●	●	●	●	
4.55	353.086	*cis-*4-Caffeoylquinic acid **(7)**	191.05, 179.03, 173.04, 135.04	●			●		
*4*.*65*	*353*.*101*	*trans-4-Caffeoylquinic acid* ***(8)***	*191*.*03*, *179*.*00*, *173*.*00*, *135*.*03*	*○*	*○*		*○*		
5.22	355.091	Feruloylglycoside **(4)**	193.04			●			●
*5*.*36*	*355*.*099*	*Feruloylglycoside* ***(4)***	*193*.*06*	*○*					
5.47	353.077	*cis*-5-Caffeoylquinic acid **(9)**	191.05, 135.04	●	●	●		●	
5.55	335.051	3-Caffeoylshikimic acid **(10)**	178.93			●			
5.77	337.194	*5-p*-Coumaroylquinic acid **(11)**	191.05	●	●	●	●		
6.03	335.071	4-Caffeoylshikimic acid **(12)**	179.03, 160.02, 135.03	●	●	●			●
6.28	348.176	Unknown	281.13, 178.92						●
6.69	367.104	5-Feruloylquinic acid **(13)**	191.05	●	●	●			
6.87	443.150	Unknown	348.17, 189.02						●
6.93	337.085	Unknown	195.06, 180.04		●	●			
*7*.*37*	*444*.*145*	*Coumaroyltyramine glycoside isomer 1* ***(14A/B)***	*282*.*10*			*○*			
7.49	587.236	Unknown	475.16, 367.13, 206.99, 175.95						●
7.54	319.078	*p*-Coumaroylshikimic acid **(15)**	163.01, 119.05	●	●	●			
7.66	474.181	Feruloyltyramine glycoside isomer 1 **(16A/B)**	312.11, 178.05	●	●	●		●	
7.84	444.161	Coumaroyltyramine glycoside isomer 2 **(14A/B)**	282.10	●		●			
*7*.*95*	*474*.*184*	*Feruloyltyramine glycoside isomer 2* ***(16A/B)***	*312*.*11*, *178*.*05*	*○*	●	●		●	
8.09	474.175	Feruloyltyramine glycoside isomer 3 **(16A/ B)**	312.11, 178.05		●				
8.18	474.172	Feruloyltyramine glycoside isomer 4 **(16A/B)**	312.11, 178.05	●	●				
*8*.*31*	*504*.*182*	*Feruloyl-3-methoxytyramine glycoside (****17A/ B****)*	*342*.*13*, *178*.*10*	*○*	*○*		●		
8.36	515.161	3,4-*di*Caffeoylquinic acid **(18)**	353.08, 191.05, 179.03, 173.04, 135.04	●	●	●		●	
*8*.*64*	*546*.*194*	*Feruloyl-3-methoxytyramine conjugate isomer 1* ***(19A/B)***	*342*.*13*, *178*.*04*	*○*	*○*	●	●	●	
8.74	515.120	4,5-*di*Caffeoylquinic acid **(20)**	353.08, 191.05, 179.03, 173.04, 135.04	●	●	●			
8.80	546.196	Feruloyl-3-methoxytyramine conjugate isomer 1 **(19A/B)**	342.13, 178.04	●	●	●		●	
9.03	499.188	Unknown	273.16, 193.06						●

### Metabolite changes induced by defence-related phytohormones

Defence signalling networks include phytohormones (*e*.*g*. SA, JA, ET, ABA, IAA) which regulate the launching of a specific immune response [1,20]. These molecules are known to be activators or modulators of plant defence signalling and, in some cases, the activated plant defence pathways have been reported [1,2,20] and defence genes and—proteins associated with a particular signalling molecule or dependent-defence pathway identified [24,25]. In this study, three phytohormones namely ABA, MJ and SA were used to study the associated triggered metabolite responses. The annotated metabolites are listed in Table 1.

### Metabolite annotation and characterisation

Plants produce an enormous variety of secondary metabolites and consequently correct metabolite annotation is difficult and time-consuming due to scarcity of authentic standards and limited database entries. Recent advances in mass spectrometry instruments with the ability to report mass accuracy below 3 ppm, have assisted in overcoming this problem [51]. Here, Q-TOF-MS was used to profile all metabolites induced by the different plant elicitors. To prevent incorrect usage of the IUPAC number ring system, different factors from the chromatographic separation to MS were taken into consideration as reported in previous publications [52,53]. In the present study, data acquisition was done by Q-TOF-MS using an energy ramping method and a similar MS/MS metabolite identification approach as previously published by us was followed [33,54]. Below, we illustrate the annotation of hydroxycinnamic acid (HCA) amines, identified based on their fragmentation patterns obtained with MS, as an example. The other molecules and their associated diagnostic peaks are listed on Table 1 and their corresponding single ion chromatograms and spectra shown as supporting information (Figs A–H in S1 File).

**Fig 5:**
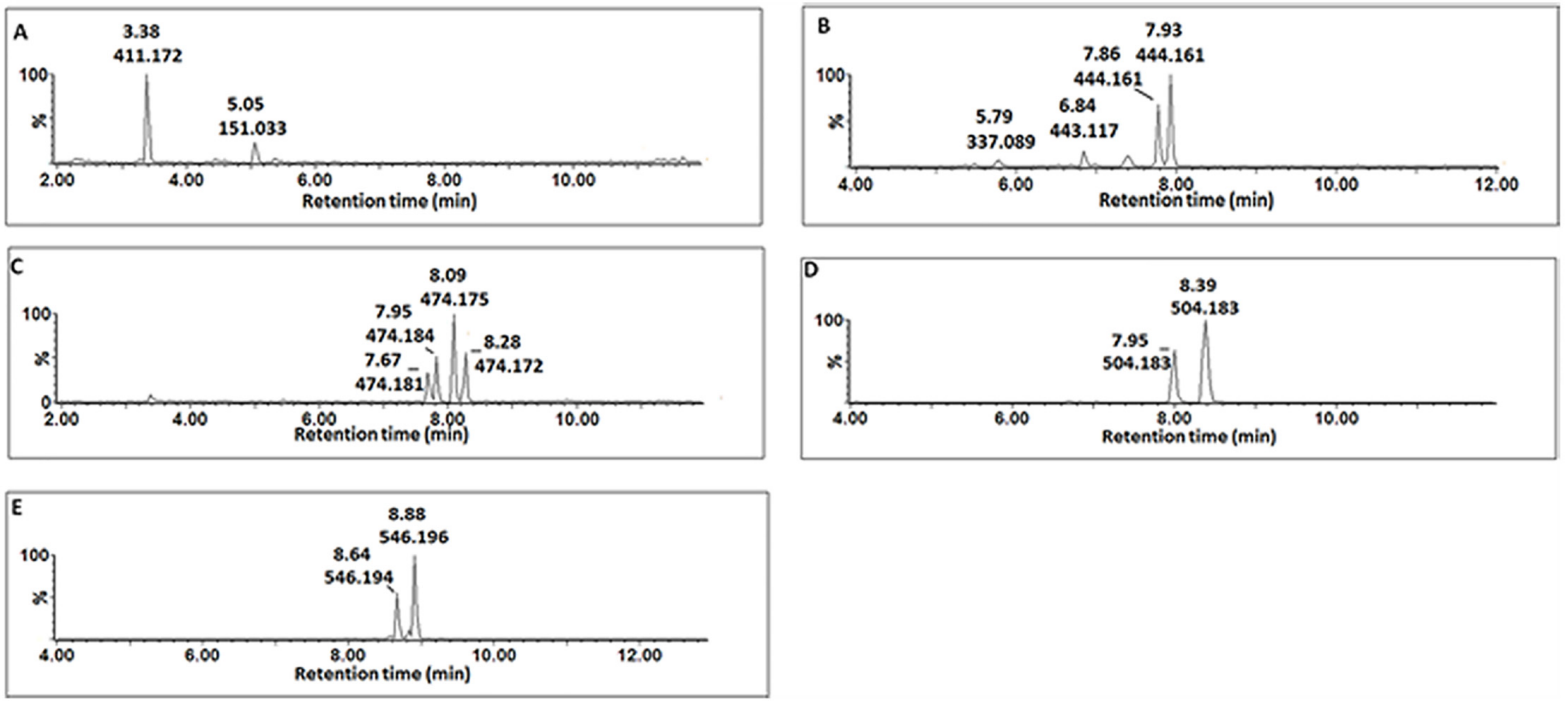
Single ion chromatograms (XIC) of UHPLC-MS/MS showing the retention times of cinnamic acid derivatives conjugated to nitrogen containing molecules. Caffeoylputrescine glycoside **(A)**, *p*-coumaroyltyramine glycoside **(B)**, feruloyltyramine glycoside **(C)**, feruloyl-3-methoxytyramine-4-glycoside **(D)** and feruloyl-3-methoxytyramine conjugate **(E).**

**Fig 6:**
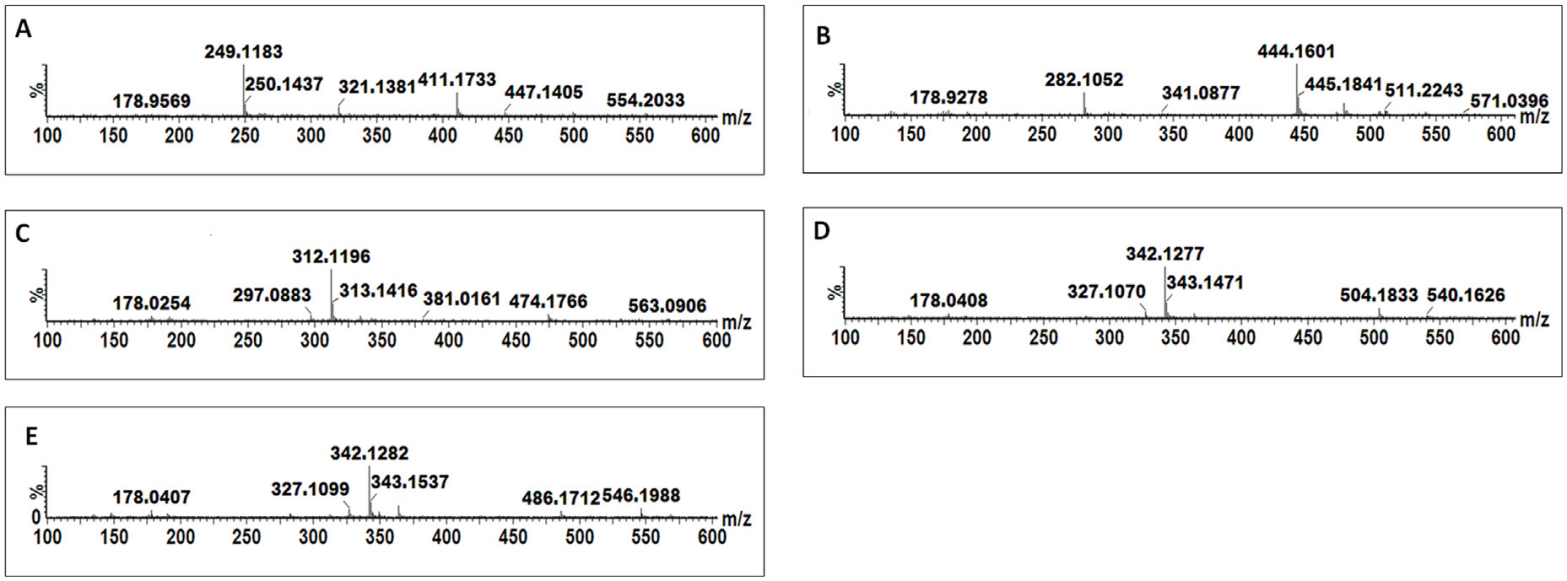
MS spectra showing fragmentation patterns of cinnamic acid derivatives conjugated to nitrogen containing molecules. Caffeoylputrescine glycoside **(A)** *p*-coumaroyltyramine glycoside **(B),** feruloyltyramine glycoside **(C)**, feruloyl-3-methoxytyramine-4-glycoside **(D)** and feruloyl-3-methoxytyramine conjugate **(E)**.

A peak at *m/z* 411.17 was detected in CHT-, FLG-, MJ- and SA-treated cells (Fig 5A) and MS fragmentation patterns showed peaks at *m/*z 321.14, 249.12 ([caffeoylputrescine-162 Da]^-^), 178.96 ([caffeoyl-C_4_H_12_N_2_]^-^) (Fig 6A). Based on this information the molecule was annotated and putatively identified as caffeoylputrescine glycoside **(1)**.

A pseudomolecular peak at *m/z* 444.79 was detected in CHT and LPS treated cells (Fig 5B). The peak produced a base peak at *m/z* 282.10 ([*p*-coumaroyl-H-162 Da]^-^) by the loss of a glycosyl residue (Fig 6B). The peak at *m/z* 282.10 corresponds to *p*-coumaroyltyramine; hence the molecule was annotated as a *p*-coumaroyltyramine glycoside isomer **(14A/B)**.

Four peaks at *m/z* 474 were detected and their accumulation was found to differ among the inducers (either one or all four were significantly altered by the different treatments) (Fig 5C). Also, the peaks were found at different retention times (Fig 5C), which indicates that they could either be *stereo*- or *regio*-isomers of each other. The MS fragments of these peaks produce base peaks at *m/z* 312.11 ([feruloyl-tyramine-H-160 Da]^-^) after loss of a glycosyl residue and a peak at *m/z* 178.03 ([feruloyl-tyramine-H-]^-^), indicating a tyramine residue loss (Fig 6C). Based on this information, the molecules were annotated as feruloyltyramine glycosides **(15A/B)**.

Two peaks with pseudomolecular ion peak at *m/z* 504.18 were significantly induced by MJ and SA (Fig 5D). Their MS fragments produced distinctive ions at *m/z* 342.13 ([feruloyl-3-methoxytyramine-H-162 Da]^-^) as a result of glycosyl residue loss and 178.04 ([feruloyl-H-CH_3_-tyramine]^-^) after losing the feruloyl methoxy group and 3-methoxytyramine residue (Fig 6D). Based on this information, this molecule was annotated as feruloyl-3-methoxytyramine glycoside **(17A/B).**

Lastly, two peaks at *m/z* 546.18 were detected in all MAMPs- and MJ-treated cells (Fig 5E). Their MS fragmentation produced base peaks at *m/z* 342.13 ([feruloyl-3-methoxytyramine-H-203 Da]^-^), 327.11 ([feruloyltyramine-H-CH_3_]^-^) due to the loss of a methoxy group and 178.04 ([feruloyl-H-CH_3_-tyramine]^-^) after losing the feruloyl methoxy group and tyramine residue (Fig 6E). From the information the molecule was annotated as a feruloyl-3-methoxytyramine conjugate with an unknown R group of m/z = 204 **(19A/B)**.

Where the HCAs were found to be conjugated to tyramine, (*e*.*g*. reported as feruloyltyramine glycoside **(15A/B)**, *p*-coumaroyltyramine glycoside **(14A/B)** and feruloyl-3-methoxytyramine conjugate **(19A/B),** the glycosyl group can be attached either on the cinnamic acid or the tyramine. As seen in Fig 5D and 5E, the molecules have the same *m/z* but different Rts which could be due to the position of the sugar, as well as *trans-* or *cis-*isomerisation of the cinnamic acids.

### Comparison of perturbed metabolites in response to MAMP perception

Comparative profiling of the biomarkers induced by MAMPs (LPS, CHT and FLG22) and phytohormones (ABA, MJ and SA) were performed to find shared metabolites that can be used to answer the following questions: (1) do these elicitors stimulate similar metabolic pathways and (2) can the shared metabolites be used to investigate MAMP elicitor phytohormone-dependent signalling pathways leading to a primed state.

**Fig 7:**
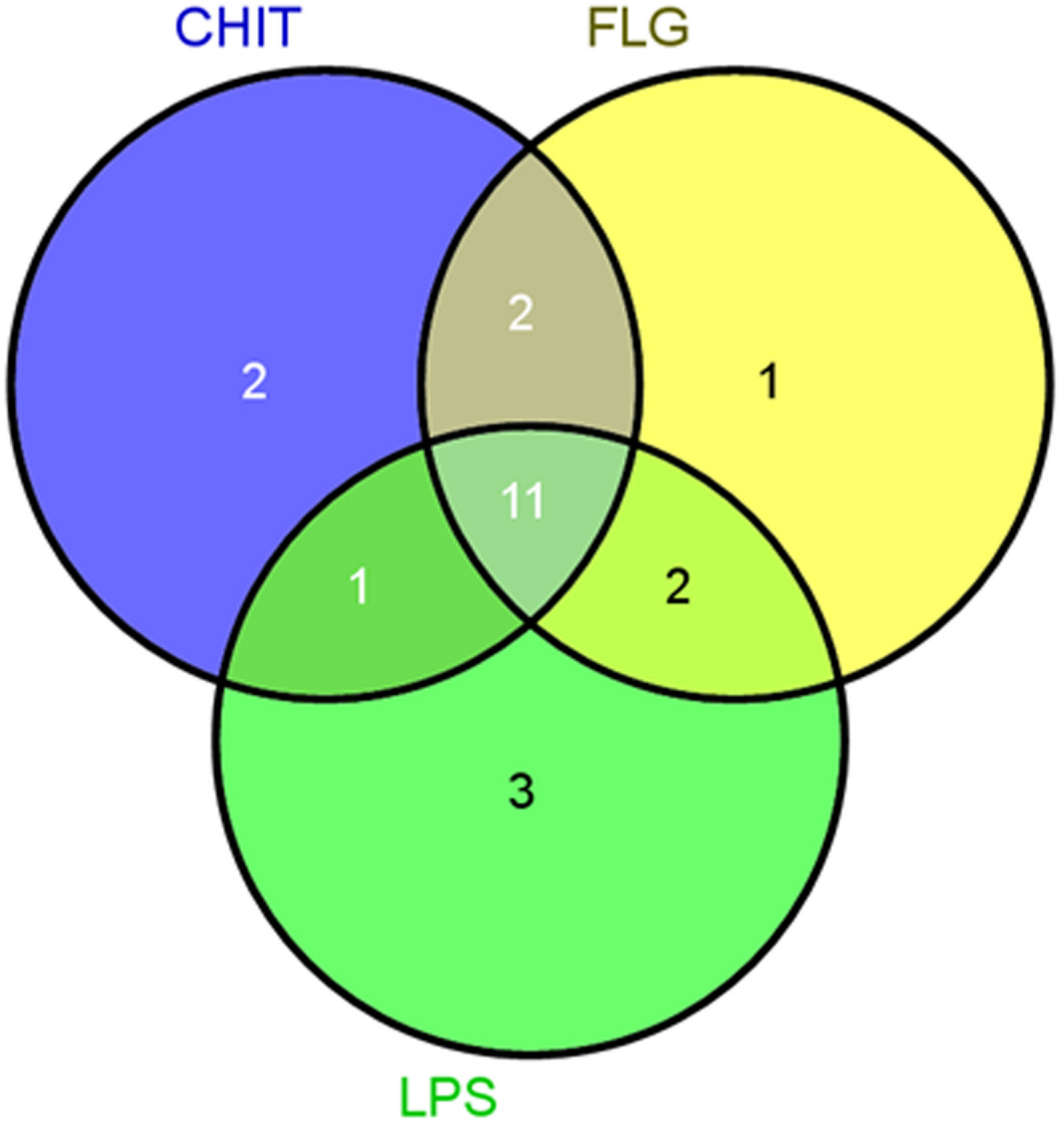
Venn diagram of metabolites positively correlated to the lipopolysaccharide (LPS), chitosan (CHT) and flagellin peptide-22 (FLG) treatment of tobacco cells. The diagram shows overlapping and distinct metabolites indicated by the numbers in the intersections and circles respectively. The specific metabolites are indicated in Table 1.

To achieve this, Venn diagrams were created. Firstly, the MAMP–elicited responses were compared among themselves and the following was observed (Table 1): eleven metabolites **(2, 6, 8, 11, 12, 13, 15, 16A/B, 18, 19A/B, 20)** were found common to all three inducers. Thirteen metabolites **(1, 2, 6, 9, 11, 12, 13, 15, 16A/B, 16A/B, 18, 19A/B, 20)** were common between CHT- and FLG-treated cells. Thirteen metabolites **(2, 6, 9, 11, 12, 13, Unknown, 15, 16/B, 16A/B, 18, 19A/B, 20)** were shared between FLG- and LPS-treated cells, and twelve metabolites **(2, 6, 9, 11, 12, 13, 14A/B, 15, 16A/B, 18, 19A/B, 20)** were common in CHT- and LPS-treated cells. CHT-treated cells had two unique metabolites **(3** and **5)**, FLG-treated cells had one unique metabolites **(16A/B)**, and cells treated with LPS had three unique metabolites **(4, 10** and **19A/B)**. These findings are schematically illustrated in Fig 7.

Although it is known that MAMP-triggered responses lead to activation of defence pathways, there are very few studies where the responses have been studied at a metabolome level. The use of a specific type of secondary metabolite pathway for defence is associated with the applicable genus or sometimes species (*e*.*g*. glucosinolates, terpenes, isoflavanoids, *etc*.). In the case of the *Solanaceae*, molecules associated with the phenylpropanoid pathway are often utilised [55].

The accumulation of phenylpropanoid derivatives in *Cocos nucifera* cell suspensions stimulated with CHT was similarly reported [56]. Increased activity of phenylpropanoid pathway enzymes such as phenylalanine ammonia lyase (PAL), as well as *p*-coumaroyl-CoA ligase and *p*-hydroxybenzaldehyde dehydrogenase, explained the accumulation of such compounds in CHT-treated cells. Postharvest-treatment of potatoes with CHT induced resistance against *Fusarium sulphureum* and also increased the PAL activity leading accumulation of flavonoids and lignin [48].

LPS pretreatment on pepper leaves led to the induction of an immune response against bacterial infection. This response was associated with the accumulation of phenolic conjugates such as feruloyltyramine and coumaroyltyramine [57,58]. These molecules were proposed to play a role in pathogen resistance when incorporated into the cell wall. Interestingly, the accumulation of the two metabolites was associated with increased tyramine hydroxycinnamoyl tranferase activity but with no increase in PAL transcripts [57].

In contrast to LPS and CHT, FLG is one of the well-documented MAMPs with no metabolic data reported regarding its induced response.

From Fig 7 the altered metabolomes triggered by the MAMPs have ten metabolites in common which indicates that they all act *via* the phenylpropanoid pathway. The distinctive metabolites or metabolites common between the MAMPs indicates that even though the activation of the general phenylpropanoid pathway leading to defence is the same, the metabolite composition, as reflected in the unique features/biomarkers that are positively or negatively correlated to the treatments, is somehow still partially dependent on the unique physicochemical properties of the three MAMPs and aspects related to their perception.

Interestingly, some metabolites that were indicated by the OPLS-DA analysis as negatively correlated with the MAMP treatments, were annotated as isomers of positively correlated metabolites (Table 1). Cinnamic acid derivatives exhibit a wide structural complexity, mainly due to positional—and geometrical (*cis vs*. *trans*) isomerism and conjugation [52, 59, 60]. We recently showed and argued the involvement of both regional and geometrical isomerism to be a strategy deployed by plants to maximize the defensive metabolites through isomerism [61]. The data suggests that different HCA isomers are utilized differently, with the down-regulated metabolites probably acting as preformed reserves which is used to mitigate the effect imposed by various stress—and signaling molecules. In turn, the inducible / up-regulated metabolites serve to replenish the depleted pools and to mount a stronger response when needed (discussed below).

### Comparison of induced metabolites in response to MAMP elicitation and phytohormone-induced metabolites

**Fig 8:**
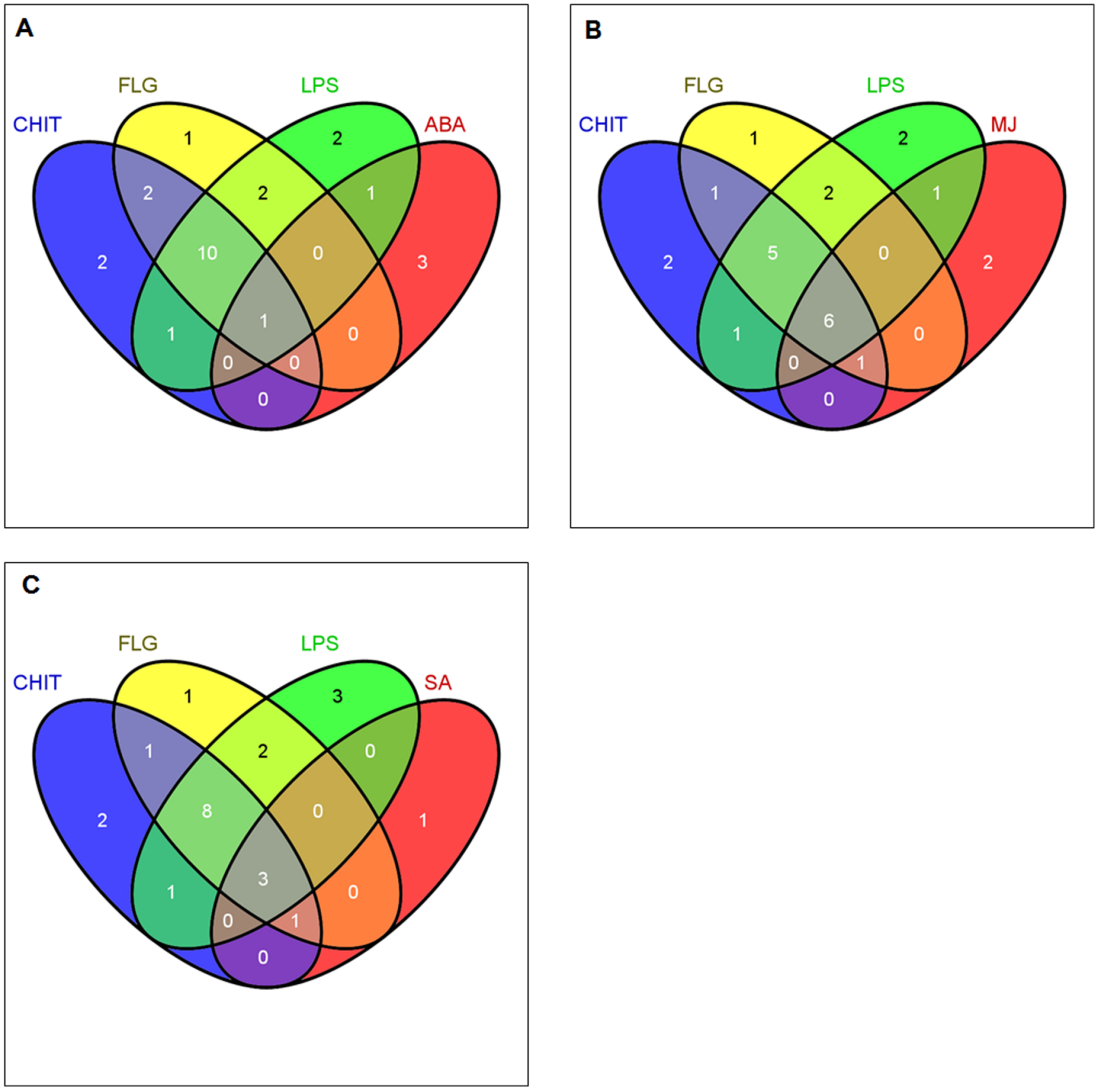
Venn diagrams of induced metabolites in elicited tobacco cells. Comparison of metabolites induced by MAMPs (lipopolysaccharides, chitosan and flagellin peptide-22 to (**A**), absciscic acid, (**B**) methyljasmonate and (**C**) salicylic acid. The diagrams show overlapping and distinct metabolites indicated by the numbers in the intersections and circles respectively.

The comparison of induced metabolites in response to MAMP elicitation was extended to include the metabolites induced by ABA, MJ and SA (Fig 8) to investigate if a connection to phytohormone-dependent defence pathways can be discerned using a metabolomic approach.

#### Absiscic acid-induced metabolites

Modulation of both primary and secondary metabolites is one priming mechanism ascribed to ABA [62]. Here, only one metabolite, (**12,** 4-caffeoylshikimic acid), of the 11 metabolites common to MAMPs was present in the ABA group (Fig 8A). One of the three metabolites only found in LPS-treated cells (Table 1 and Fig 7), (**4**, feruloylglycoside), was found in the ABA group. Otherwise the pattern in Fig 7 did not change for CHT- and FLG-treated cells when compared to ABA-treated cells. This indicates that the action mechanisms of LPS, CHT and FLG do not overlap with that of ABA. ABA is more involved in abiotic stress responses, although recent research does indicate some overlap with biotic stress responses [63]. Three unkown molecules were found exclusively in ABA-treated cells.

The two cinnamic acid derivatives (**4,** feruloylglycoside and **12,** 4-caffeoylshikimic acid) could suggest that ABA does have some effect on the phenylpropanoid pathway, however, this assumption is based on only two molecules and should be interpreted with care. Since the inhibitory effect on lipoxygenase (LOX) suggests that ABA induces resistance by suppressing the octadecanoid pathway. Application of ABA on *Orthosiphon stamineus* (Java tea) foliage lead to the generation of H_2_O_2_, O_2_^.-^ and enhanced PAL activity, and accumulation of phenolics and soluble sugars [64].

### Methyljasmonate-induced metabolites

Six metabolites (**6, 9, 12, 18** and **19A/B,** *trans*-5-caffeoylquinic acid, *cis*-5-caffeoylquinic acid, 4-caffeoylshikimic acid, 3,4-*di*caffeoylquinic acid and feruloyl-3-methoxytyramine conjugate isomers) of eleven common amongst the MAMP-treated cells were also found in MJ-treated cells (Fig 8B). These findings show that MJ treatment leads to activation of the phenylpropanoid pathway and suggests that the action mechanism of the MAMPs involves activation of the MJ-dependent defence signalling pathway. One of the three compounds found only in LPS-treated cells (**19A/B,** feruloyl-3-methoxytyramine conjugate) was found in MJ treated cells as well, and one metabolite (**1,** caffeoylputrescine glycoside) of the two metabolites common in CHT- and FLG-treated cells was found in MJ-treated cells. Overlapping metabolites between CHT- and FLG-treated cells and CHT- and LPS-treated cells (Fig 7) remained the same when compared to the different phytohormones (Fig 8).

Jasmonates have been reported to affect a number of metabolic pathways in plants leading to production of secondary metabolites [65] which later contribute to plant resistance. Treatment of barley seedlings with MJ lead to the alteration of polyamine metabolism and primed the plants against powdery mildew infection. This priming was also associated with increased activities of defence enzymes such as PAL and peroxidases [66]. The increase of PAL activity indicated the stimulation of the phenylpropanoid pathway leading to accumulation of phenolics. The production of HCA amides has been reported as a response against fungal infection [66], and the accumulation of free polyamines and cinnamic acid derivatives in MJ-treated plants could explain the production of these conjugates.

### Salicylic acid-induced metabolites

Lastly, the induced metabolites in response to MAMP priming were compared to those induced by SA. Considering Figs 7 and 8C, four metabolites (**2, 6, 11** and **20,** 3-caffeoylquinic acid, *trans*-5-caffeoylquinic acid, 5-*p*-coumaroylquinic and 4,5-*di-*caffeoylquinic acid), were also found in SA-treated cells. This also confirms (at a metabolite level) that SA stimulates the phenylpropanoid pathways. Furthermore, it also indicates that the defence signalling pathway(s) leading to defence activation by MAMP molecules involves SA. One molecule (**1**, caffeoylputrescine glycoside) of the two molecules common in CHT- and FLG-treated cells was present in SA-treated cells. The pattern in Fig 8C showing overlapping metabolites between FLG- and LPS-treated cells, and the overlapping metabolites between CHT- and LPS-treated cells did not changed from the one observed in Fig 7. Feruloyl-3-methoxytyramine glycoside isomer (**17A/B**) was only present in SA-treated cells.

Priming by SA results in the accumulation of PAL gene transcripts and enhanced activity of this enzyme which, in turns, leads to accumulation of phenolics and lignin [67], and other derivatives of the phenylpropanoid pathway, associated with aspects of plant priming.

### General discussion: cinnamic acid conjugates as biomarkers of an induced defensive state

It has been hypothesised that priming developed during evolution to assist plants to rapidly adapt to new situations. The advantage of priming is that it offers the plant an enhanced protection without the costs of constitutively expressing their defence genes. However, the molecular mechanisms and metabolic basis of this stress-imprinting on plant immunity is not fully elucidated, and may vary between plants from different families. In the case of the *Solanaceae* it could depend on the controlled, dynamic balance between biosynthesis and degradation of phenolic compounds. The phenylpropanoid pathway with HCA intermediates is an important pathway, linked to resistance, for the production of different phenolics in free or conjugated forms.

Cinnamic acid (and its hydroxylated and methoxylated derivatives) originates from phenylalanine in response to stress-induced increases in PAL activity. In turn, phenylalanine is synthesized via the shikimate pathway that also supplies the alicyclic acids dehydroquinate and dehydroshikimate. The quinic acid (QA) pool acts as a reservoir that can be reversibly injected into the main pathway [68] for esterification reactions with the HCAs. CGAs may be synthesised via the condensation of QA with p-coumaroyl-CoA by a hydroxycinnamoyl CoA quinate hydroxycinnamoyl transferase and p-coumaroyl ester 3’-hydroxylase [69]. In comparison, the other related derivatives are synthesised via the condensation of HCA derivatives with either a sugar, polyamines (putrescine and spermidine) or tyramine. The HCA derivatives, CGAs and related conjugates can furthermore be glycosylated, for possible storage, an observation that is in line with [70].

The knowledge of phenolics as defence molecules has long been realised [70], and the involvement of HCAs and chlorogenic acids (CGAs) in plant defence have been reported elsewhere [55]. CGAs and related derivatives have been identified as phytoanticipins [71] and resistance biomarkers [72–74], conferring resistance to herbivore feeding and pathogen infection [75,76]. CGAs have also been reported to inhibit enzymes used by the pathogens to infect plants [77]. CGA oxidation by phenoloxidases yield quinones and chlorogenoquinones. The resulting quinones have the ability to react with amino- and sulfhydryl groups of proteins, and thus could lead to inhibition of pathogen-associated enzymes [78]. Classical examples of CGAs acting as non-antimicrobial defence compounds, but interfering with infection processes, include the inhibition of appressorium formation [79], inhibition of melanin synthesis in the peach: *Monilinia laxa* interaction [80], and countering fungal toxin synthesis in the tomato: *Alternaria alternata* interaction [81].

CGAs represent only one form of conjugated HCAs. In addition to conjugation through an ester linkage, conjugates with tyramine and polyamines through amide bond linkages were also found in this metabolomic study. In the context of priming, pre-treatment of pepper leaves with LPS was associated with accumulation of feruloyltyramine and coumaroyltyramine [57]. Recently, it was reported that the fungal-derived MAMP, ergosterol, induced dynamic changes in *N*. *tabacum* cells, and these changes were associated with CGA (caffeoylquinic acid) and other cinnamic acids conjugates such as 1-*O*-sinapoyl-beta-glucose, 4-coumaroylshikimic acid, caffeoylshikimic acid, cinnamoyltyramine, *N*-caffeoylputrescine and sinapoyltyramine [15].

**Fig 9:**
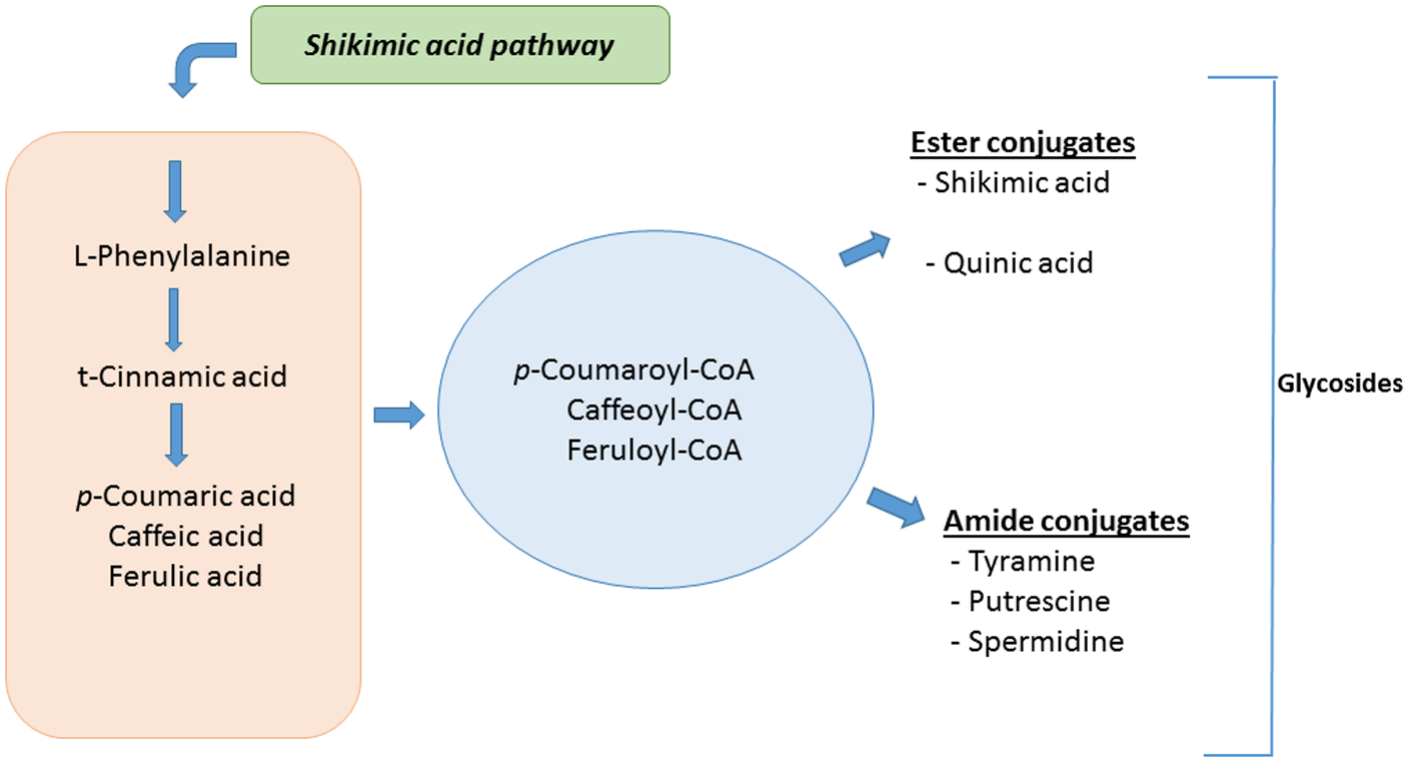
A role for hydroxycinnamic acids and—conjugates as mediators of priming in tobacco cells. Overview of the metabolites and biochemical pathways highlighted by metabolomic analysis of *Nicotiana tabacum* cells treated with lipopolysaccharides, chitosan and flagellin (flg22). The origin of these priming-associated secondary metabolites lies in the primary metabolites phenylalanine, tyrosine, arginine, proline, glutamic acid and glucose. The accumulation of the ester—and amide conjugates of hydroxycinnamic acids (HCAs) and their rapid hydrolysis upon pathogen attack can allow the plant to launch a more rapid and intense response upon pathogen attack.

The accumulation of free and conjugated HCAs thus seems to be part of the priming mechanism in solanaceous plants (Fig 9), contributing to an enhanced defensive capacity. Reversal of the biosynthetic reactions / hydrolysis of the HCA conjugates upon attempted pathogen attack would rapidly release the HCA derivatives (*p*-coumaric-, caffeic- and ferulic acids), which can then be utilised in various aspects of chemical defences such as phytoalexin synthesis or as lignin precursors for cell wall strengthening [82]. This inter-conversional link between phytoanticipins and phytoalexins offers a plausible explanation for the accumulation of ester and amide conjugates of HCAs during priming. Plants that have been primed to accumulate high levels of these HCA derivatives are enabled to launch a stronger and faster defence response upon subsequent infections.

## Conclusion

Metabolome change is a strategy used by a host to cope with changes in the external and internal environment [83]. In the case of the plant innate immune system, exogenous metabolites can reprogram the metabolic strategy to adapt to such changes [7–9, 21]. The results demonstrate the power of metabolomic approaches in investigating the responses of plant cells to individual microbe/pathogen-derived ‘non-self’ molecules. Considerable overlap between the biomarkers identified in response to LPS, chitosan and flg22 indicates that the individual MAMPs trigger signal transduction pathways that converge to result in similar metabolomes in support of priming and/or MTI. It was found that phytohormone-dependent signalling pathways, assessed at a metabolic level, could be associated with the responses to the different MAMPs. Here, it was observed that the triggering of defence responses by lipopolysaccharides, chitosan and the flg22 peptide from flagellin is modulated by both SA as well as MJ, with ABA not playing a significant role. The results obtained in this study therefore contribute to a better understanding of the action mechanisms of the three MAMPs that can be utilised to design novel priming-related protection strategies against crop losses due to pathogen attack.

## Supporting Information

S1 File **A Fig. *p*-Coumaroylshikimic acid**. Extracted single ion chromatograms (XIC) of UHPLC-MS/MS showing the retention time (A) and the corresponding MS spectrum (B). **B Fig. Caffeoylshikimic acid (CSA) isomers.** Extracted single ion chromatograms (XIC) of UHPLC-MS/MS data showing the retention times (A) and the corresponding MS fragmentation patterns (B and C). **C Fig. *p*-Coumaroylquinic acid (*p*-CQA)**. Extracted single ion chromatograms (XIC) of UHPLC-MS/MS data showing the retention times (A) and the corresponding MS fragmentation pattern (B). **D Fig. Feruloylglycoside**. Extracted single ion chromatograms (XIC) of UHPLC-MS/MS data showing the retention times (A) and the corresponding MS fragmentation pattern (B). **E Fig. Chlorogenic acids-1.** Single ion chromatograms of *mono* (A) and *di-*acylated chlorogenic acids (B), and also chlorogenic acid glycosides at Rt 3.85 and 5.04. **F Fig. Chlorogenic acids-2**. MS spectra showing fragmentation patterns of 3-CQA (A), 4-CQA (B), *cis*/*trans*-5-CQA (C), 3,4-diCQA (D) and 4,5-diCQA (E). **G Fig. 5-Feruloylquinic acid.** Extracted single ion chromatograms (XIC) of UHPLC-MS/MS data showing the retention times (A) and the corresponding MS fragmentation pattern (B). **H Fig. Caffeoyl glucosyl quinic acids.** MS spectra showing fragmentation patterns of 3-O-(4’-O-caffeoyl glucosyl) quinic acid (A) and 5-O-(3’-O-caffeoyl glucosyl) quinic acid (B).
(DOCX)
