# Supplementary material for: Phenylpropanoid Defences in Nicotiana tabacum Cells: Overlapping Metabolomes Indicate Common Aspects to Priming Responses Induced by Lipopolysaccharides, Chitosan and Flagellin-22
Source: PLoS One. 2016 Mar 15;11(3):e0151350. doi: 10.1371/journal.pone.0151350 (PMC4792386; doi:10.1371/journal.pone.0151350)
Supplement: S1 File — A Fig. p-Coumaroylshikimic acid. Extracted single ion chromatograms (XIC) of UHPLC-MS/MS showing the retention time (A) and the corresponding MS spectrum (B). B Fig. Caffeoylshikimic acid (CSA) isomers. Extracted single ion chromatograms (XIC) of UHPLC-MS/MS data showing the retention times (A) and the corresponding MS fragmentation patterns (B and C). C Fig. p-Coumaroylquinic acid (p-CQA). Extracted single ion chromatograms (XIC) of UHPLC-MS/MS data showing the retention times (A) and the corresponding MS fragmentation pattern (B). D Fig. Feruloylglycoside. Extracted single ion chromatograms (XIC) of UHPLC-MS/MS data showing the retention times (A) and the corresponding MS fragmentation pattern (B). E Fig. Chlorogenic acids-1. Single ion chromatograms of mono (A) and di-acylated chlorogenic acids (B), and also chlorogenic acid glycosides at Rt 3.85 and 5.04. F Fig. Chlorogenic acids-2. MS spectra showing fragmentation patterns of 3-CQA (A), 4-CQA (B), cis/trans-5-CQA (C), 3,4-diCQA (D) and 4,5-diCQA (E). G Fig. 5-Feruloylquinic acid. Extracted single ion chromatograms (XIC) of UHPLC-MS/MS data showing the retention times (A) and the corresponding MS fragmentation pattern (B). H Fig. Caffeoyl glucosyl quinic acids. MS spectra showing fragmentation patterns of 3-O-(4’-O-caffeoyl glucosyl) quinic acid (A) and 5-O-(3’-O-caffeoyl glucosyl) quinic acid (B). (DOCX) [file pone.0151350.s001.docx]

**Supporting Information.**

**File S1:**

**A Fig.** ***p*-Coumaroylshikimic acid**. Extracted single ion chromatograms (XIC) of UHPLC-MS/MS showing the retention time (A) and the corresponding MS spectrum (B).

**B Fig.** **Caffeoylshikimic acid (CSA) isomers.** Extracted single ion chromatograms (XIC) of UHPLC-MS/MS data showing the retention times (A) and the corresponding MS fragmentation patterns (B and C).

**C Fig.** ***p*-Coumaroylquinic acid (*p*-CQA)**. Extracted single ion chromatograms (XIC) of UHPLC-MS/MS data showing the retention times (A) and the corresponding MS fragmentation pattern (B).

**D Fig.** **Feruloylglycoside**. Extracted single ion chromatograms (XIC) of UHPLC-MS/MS data showing the retention times (A) and the corresponding MS fragmentation pattern (B).

**E Fig.** **Chlorogenic acids-1.** Single ion chromatograms of *mono* (A) and *di-*acylated chlorogenic acids (B), and also chlorogenic acid glycosides at Rt 3.85 and 5.04.

**F Fig.** **Chlorogenic acids-2**. MS spectra showing fragmentation patterns of 3-CQA (A), 4-CQA (B), *cis*/*trans*-5-CQA (C), 3,4-diCQA (D) and 4,5-diCQA (E).

**G Fig.** **5-Feruloylquinic acid.** Extracted single ion chromatograms (XIC) of UHPLC-MS/MS data showing the retention times (A) and the corresponding MS fragmentation pattern (B).

**H Fig.** **Caffeoyl glucosyl quinic acids.** MS spectra showing fragmentation patterns of 3-O-(4’-O-caffeoyl glucosyl) quinic acid (A) and 5-O-(3’-O-caffeoyl glucosyl) quinic acid (B).


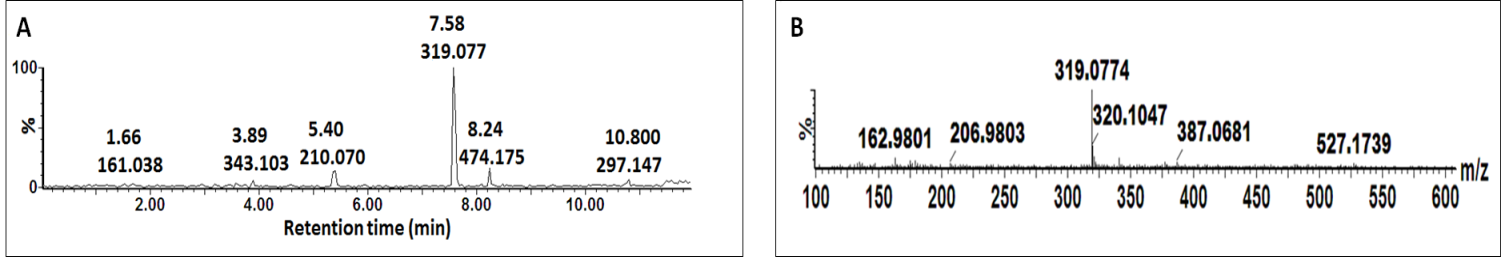


**A Fig. *p*-Coumaroylshikimic acid**. Extracted single ion chromatograms (XIC) of UHPLC-MS/MS showing the retention time (A) and the corresponding MS spectrum (B).


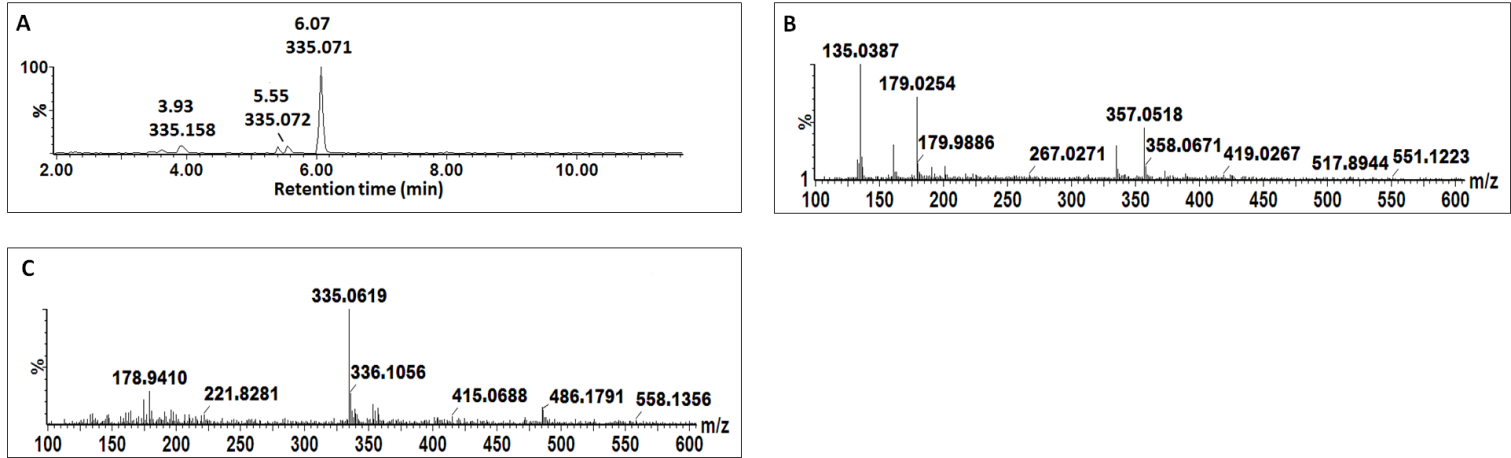


**B Fig.** **Caffeoylshikimic acid (CSA) isomers.** Extracted single ion chromatograms (XIC) of UHPLC-MS/MS data showing the retention times (A) and the corresponding MS fragmentation patterns (B and C).


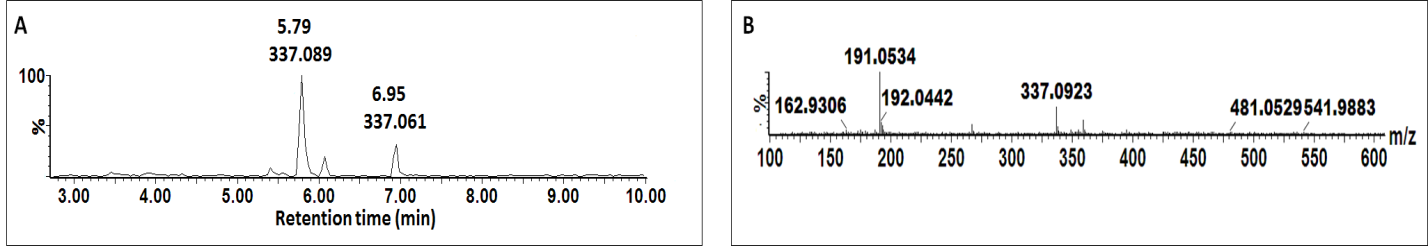


**C Fig.** ***p*-Coumaroylquinic acid (*p*-CQA)**. Extracted single ion chromatograms (XIC) of UHPLC-MS/MS data showing the retention times (A) and the corresponding MS fragmentation pattern (B).


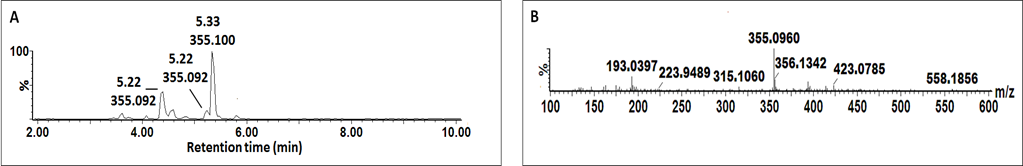


**D Fig.** **Feruloylglycoside**. Extracted single ion chromatograms (XIC) of UHPLC-MS/MS data showing the retention times (A) and the corresponding MS fragmentation pattern (B).


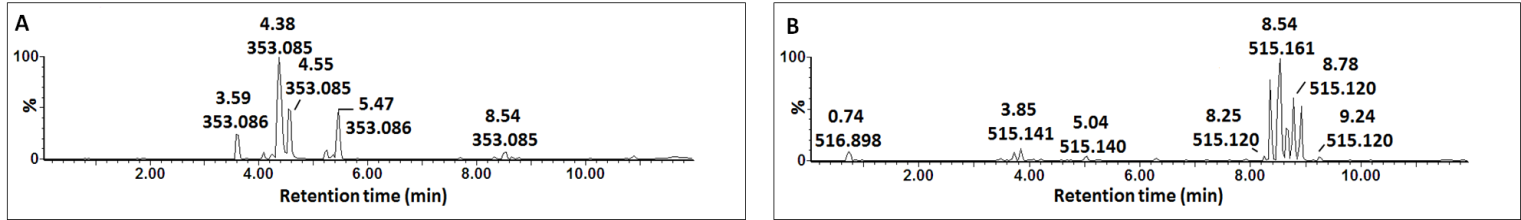


**E Fig.** **Chlorogenic acids-1.** Single ion chromatograms of *mono* (A) and *di-*acylated chlorogenic acids (B), and also chlorogenic acid glycosides at Rt 3.85 and 5.04.

**
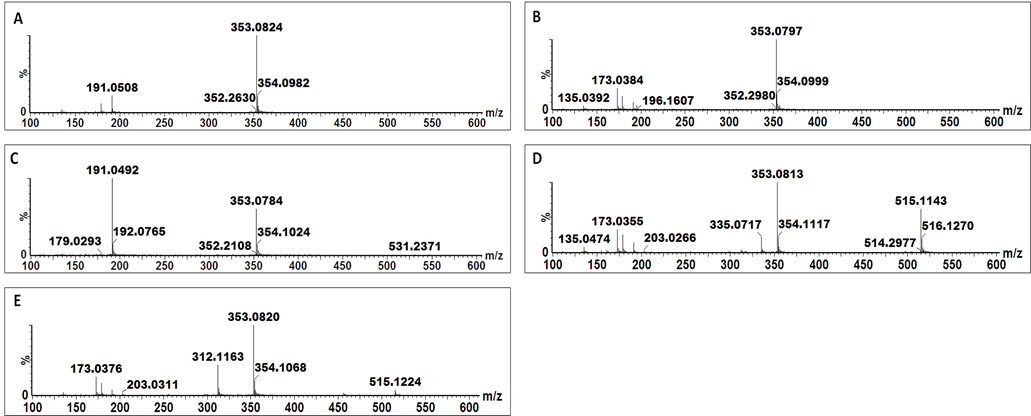
**

**F Fig.** **Chlorogenic acids-2**. MS spectra showing fragmentation patterns of 3-CQA (A), 4-CQA (B), *cis*/*trans*-5-CQA (C), 3,4-diCQA (D) and 4,5-diCQA (E).


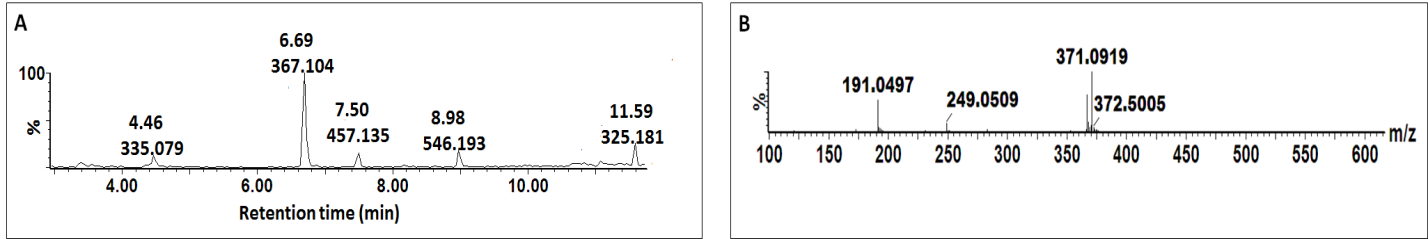


**G Fig.** **5-Feruloylquinic acid.** Extracted single ion chromatograms (XIC) of UHPLC-MS/MS data showing the retention times (A) and the corresponding MS fragmentation pattern (B).


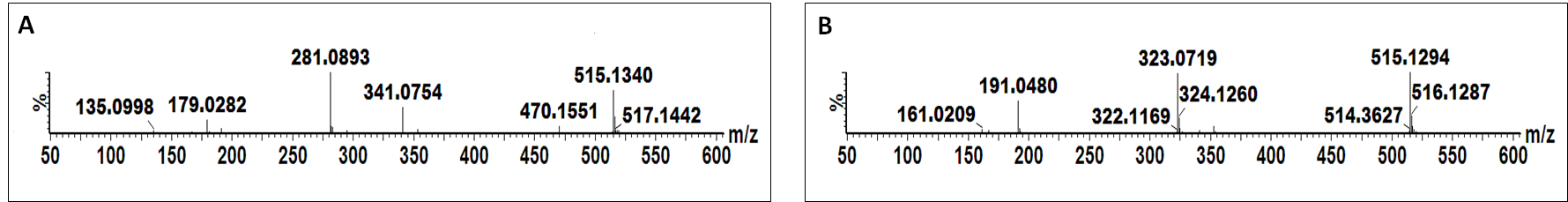


**H Fig.** **Caffeoyl glucosyl quinic acids.** MS spectra showing fragmentation patterns of 3-O-(4’-O-caffeoyl glucosyl) quinic acid (A) and 5-O-(3’-O-caffeoyl glucosyl) quinic acid (B).
